# Neuromuscular electrical stimulation prevents skeletal muscle dysfunction in adjuvant-induced arthritis rat

**DOI:** 10.1371/journal.pone.0179925

**Published:** 2017-06-21

**Authors:** Koichi Himori, Daisuke Tatebayashi, Keita Kanzaki, Masanobu Wada, Håkan Westerblad, Johanna T. Lanner, Takashi Yamada

**Affiliations:** 1 Graduate School of Health Sciences, Sapporo Medical University, Sapporo, Japan; 2 Faculty of Health and Welfare Science, Okayama Prefectural University, Soja, Okayama, Japan; 3 Graduate School of Integrated Arts and Sciences, Hiroshima University, Higashi Hiroshima, Japan; 4 Department of Physiology and Pharmacology, Karolinska Institutet, Stockholm, Sweden; University of Debrecen, HUNGARY

## Abstract

Skeletal muscle weakness is a prominent feature in patients with rheumatoid arthritis (RA). In this study, we investigated whether neuromuscular electrical stimulation (NMES) training protects against skeletal muscle dysfunction in rats with adjuvant-induced arthritis (AIA). AIA was produced by intraarticular injection of complete Freund’s adjuvant into the knees of Wistar rats. For NMES training, dorsiflexor muscles were stimulated via a surface electrode (0.5 ms pulse, 50 Hz, 2 s on/4 s off). NMES training was performed every other day for three weeks and consisted of three sets produced at three min intervals. In each set, the electrical current was set to achieve 60% of the initial maximum isometric torque and the current was progressively increased to maintain this torque; stimulation was stopped when the 60% torque could no longer be maintained. After the intervention period, extensor digitorum longus (EDL) muscles were excised and used for physiological and biochemical analyses. There was a reduction in specific force production (i.e. force per cross-sectional area) in AIA EDL muscles, which was accompanied by aggregation of the myofibrillar proteins actin and desmin. Moreover, the protein expressions of the pro-oxidative enzymes NADPH oxidase, neuronal nitric oxide synthase, p62, and the ratio of the autophagosome marker LC3bII/LC3bI were increased in AIA EDL muscles. NMES training prevented all these AIA-induced alterations. The present data suggest that NMES training prevents AIA-induced skeletal muscle weakness presumably by counteracting the formation of actin and desmin aggregates. Thus, NMES training can be an effective treatment for muscle dysfunction in patients with RA.

## Introduction

Rheumatoid cachexia occurs in approximately 10–50% of patients with rheumatoid arthritis (RA) and is characterized by the loss of muscle strength [1, 2]. Importantly, Helliwell et al. [3] reported a 60% reduction in grip strength despite only a 10% reduction in cross-sectional area of forearm muscles in RA patients. This suggests that reductions in specific force (i.e. force per cross-sectional area) as well as muscle atrophy contribute to muscle weakness in patients with RA. Indeed, reduction in maximal specific force was observed in both fast-twitch extensor digitorum longus (EDL) and flexor digitorum brevis (FDB) and slow-twitch soleus muscles from collagen-induced arthritis (CIA) mice [4, 5] and adjuvant-induced arthritis (AIA) rats [6], both widely used models for RA.

Previously we demonstrated that the impaired ability of cross-bridges to generate force was accompanied by the redox modification of myofibrillar proteins in skeletal muscles from CIA mice [4, 5]. Moreover, treatment with antioxidant prevented the intrinsic contractile dysfunction and the aggregation of actin molecules in EDL muscles from AIA rats [6], suggesting that arthritis-induced muscle weakness is at least partly caused by redox modification of actin. In support, it has been reported that actin is more susceptible to redox stress than other proteins in the contractile machinery [7], and actin oxidation can lead to formation of aggregates and impaired myofibrillar function [8, 9]. In addition to the actin, aggregation of the intermediate filament protein desmin has been associated with impaired muscle contractility in inflammatory condition [10]. It is unknown, however, whether desmin aggregates are involved in AIA-induced muscle weakness.

The level of 3-nitrotyrosine, a footprint of peroxynitrite production, and protein expression of NADPH oxidase (NOX) 2/gp91^phox^ and neuronal nitric oxide synthetase (nNOS) were increased in AIA muscles [6]. Since peroxynitrite is formed when superoxide reacts with NO, these data suggest that increased production of nNOS-derived NO and NOX2/gp91^phox^-derived superoxide favors peroxynitrite production in AIA EDL muscles. Importantly, the exposure to peroxynitrite donor has been shown to induce protein aggregates by forming intermolecular disulfides in skeletal muscle [11].

The ubiquitin-proteasome system (UPS) plays a central role in removing misfolded proteins from the cell. Deficits in UPS proteolytic function can lead to increased steady-state levels of misfolded proteins that can aggregate [12]. Previous study has demonstrated that ubiquitinated proteins are accumulated in the cell when the proteolytic function of the proteasomes is inhibited [12]. Autophagy has been identified as a major contributor in the clearance of aggregated proteins in mammalian cells [13]. Recently, it has been shown that an impaired autophagy could be responsible for the aggregation of misfolded proteins and muscle dysfunction in aging [14] and several diseases including desmin-related cardiomyopathy [15, 16]. Importantly, induction of autophagy using pharmacological intervention [17], autophagic gene overexpression [16], and voluntary exercise [15–17] protected muscles against the toxic insults of aggregated proteins by promoting their clearance.

Physical exercise has consistently been shown to improve muscle strength in patients with RA [18]. However, in patients with severe joint damage, high-intensity muscle strength exercise accelerates joint damage [19]. Recently, neuromuscular electrical stimulation (NMES) has received attention as a rehabilitation method because even at relatively low levels of evoked force, NMES activates both fast and slow motor units and thus effectively improves muscle function [20]. However, little is known about whether NMES counteracts the muscle weakness in generalized inflammatory diseases, such as in RA patients, although some encouraging results have been presented [21].

In this study, we tested the following two principal hypotheses: the force produced by EDL muscles from AIA rats was decreased and this muscle weakness was prevented by NMES; the AIA-induced muscle weakness involved formation of actin and desmin aggregates, increased peroxynitrite production by NOX2 and nNOS, and impaired autophagy flux, and these changes were also prevented by NMES.

## Materials and methods

### Ethical approval

All animal experiments were conducted with approval of Committee on Animal Experiments of Sapporo Medical University (No. 13–092). Animal care was in accordance with institutional guidelines.

### Experimental design

To examine whether NMES training prevents AIA-induced skeletal muscle dysfunction, we performed two separate experiments.

#### Experiment 1

We first assessed the effects of NMES training on the contractility of EDL muscles in normal rats. Male Wistar rats (9 weeks old, n = 6) were supplied by Sankyo Labo Service (Sapporo, Japan). Rats were given food and water ad libitum and housed in an environmentally controlled room (24 ± 2°C) with a 12-h light-dark cycle. The right leg served as a control (CNT), and NMES training was performed on the left leg (CNT + NMES) using electrical stimulator (Nihon Kohden). Throughout the NMES training sessions, rats were anesthetized by isoflurane inhalation. Rats were placed supine on a platform and their left foot was secured in a foot plate connected to a force transducer at an angle of 60 degree plantarflexion (*see* S1 Fig). Dorsiflexor muscles, including the tibialis anterior and the EDL muscles, were stimulated via the surface electrode that was placed on the skin surface of the peroneal nerve. Placement of the electrode was confirmed when stimulation elicited full ankle dorsiflexion and toe extension. Stimulation parameters were set as follows: 50 Hz, 0.5 ms pulse duration, 2 s contraction every 4 s. Torque traces were displayed on a monitor, and the stimulation intensity was progressively increased throughout the stimulation period in order to maintain a peak torque corresponding to 60% of the initial maximum isometric torque, which was measured in every NMES training sessions. We used this kind of adjustment, because it is routinely used for strength training protocols [22]. Moreover, although supramaximal stimulation has been shown to induce strength gains in rat skeletal muscles [23], it is difficult to apply supramaximal electrical stimulation in a clinical setting due to discomfort, pain or burning sensations [24]. NMES training was terminated when the torque fell below target value despite stimulation intensity reached supramaximum voltage (30 V). Each session consisted of 3 sets at 3 minutes intervals and was carried out every other day for 3 weeks. At the completion of the NMES training, rats were killed by cervical dislocation under isoflurane anesthesia and the EDL muscles were dissected from each animal.

#### Experiment 2

To investigate whether NMES training prevents AIA-induced muscle weakness, rats (9 weeks old, n = 24) were randomly assigned into CNT (n = 8), AIA (n = 9), and AIA plus NMES training (AIA + NMES, n = 7) groups. AIA was induced in the knees by intraarticular injection of 0.2 ml of cocktail containing Freund’s incomplete adjuvant (Difco) and 2 mg *Mycobacterium butyricum* (Difco) under isoflurane anesthesia [6]. The above described ES training was started 24 h after intraarticular injection and carried out every other day for 3 weeks.

### *In vitro* force measurement

Intact EDL muscles were mounted between a force transducer (Nihon Kohden) and an adjustable holder, and superfused with Tyrode solution (mM): NaCl, 121; KCL, 5; CaCl_2_, 1.8; MgCl_2_, 0.5; NaH_2_PO_4_, 0.4, NaHCO_3_, 24; EDTA, 0.1; glucose, 5.5. The solution was bubbled with 5% CO_2_-95% O_2_, which gives an extracellular pH of 7.4, and kept at 30°C. Supramaximal, 0.5 ms monophasic rectangular pulses were applied via two platinum plate electrodes placed on each side of the muscle. Muscle length was adjusted to the length (L_0_) giving maximum tetanic force and measured with a digital caliper. The force-frequency relationship was determined by evoking tetani at different frequencies (10–120 Hz, 600 ms duration) at 1 min intervals. Control experiments confirmed that 1 min intervals are sufficient to avoid fatigue-induced changes in tetanic force production (data not shown). Absolute force was normalized to cross-sectional area, calculated as muscle weight divided by L_0_ and density (1056 kg m^-3^).

### Immunoblots

Immunoblots were performed using: anti-actin (A4700, Sigma), anti-desmin (ab32362, Abcam), NOX2/gp91^phox^ (ab31092, Abcam), anti-nNOS (610308, BD Biosciences), anti-manganese superoxide dismutase (SOD2) (06–984, Upstate), anti-catalase (C0979, Sigma), anti-p62 (ab56416, Abcam), anti-microtubule-associated protein light chain 3b (LC3b) (ab63817, Abcam), and anti-GAPDH (010–25521, Wako).

Muscle pieces were homogenized in ice-cold homogenizing buffer (40 μl/mg wet wt) consisting of (mM): Tris maleate, 10; NaF, 35; NaVO_4_, 1; 1% Triton X 100 (vol/vol), and 1 tablet of protease inhibitor cocktail (Roche) per 50 ml. To extract myofibrillar proteins, an aliquot of homogenized muscle was centrifuged at 4°C for 15 min at 14,000 g. The supernatant was discarded and the resulting myofibrillar enriched pellet was resuspended in ice-cold high-salt buffer (40 μl/mg wet wt) consisting of (mM): NaCl, 300; NaH_2_PO_4_, 100; Na_2_HPO_4_, 50; Na_4_P_2_O_7_, 10; MgCl_2_, 1; EDTA, 10; pH 6.5, and 1 tablet of protease inhibitor cocktail (Roche) per 50 ml. The protein content was determined using Bradford assay [25].

Aliquots of the whole muscle homogenates (20 μg) were diluted with SDS-sample buffer (mM): Tris/HCl, 62.5; 2% SDS (wt/vol); 10% glycerol (vol/vol); 5% 2-mercaptoethanol (vol/vol); 0.02% bromophenol blue (wt/vol). For the detection of actin, desmin, and ubiquitin, proteins (20 μg) were diluted with non-reducing Laemmli buffer (mM): urea, 4000; Tris, 250; 4% SDS (vol/vol); 20% glycerol (vol/vol); 0.02% bromophenol blue (wt/vol). Proteins were applied to a 4–15% Criterion Stain Free gel (BioRad, Hercules, CA). Gels were imaged (BioRad Stain Free imager), and then proteins were transferred onto polyvinylidine fluoride membranes. Membranes were blocked in 3% (wt/vol) non-fat milk, Tris-buffered saline containing 0.05% (vol/vol) Tween 20, followed by incubation with primary antibody, made up in 5% (wt/vol) non-fat milk overnight at 4°C. Membranes were then washed and incubated for 1 h at room temperature (~23°C) with secondary antibody (1:5000, donkey-anti-rabbit or donkey-anti-mouse, BioRad). Images of membrane were collected following exposure to chemiluminescence substrate (Millipore) using a charge-coupled device camera attached to ChemiDOC MP (BioRad), and Image Lab Software (BioRad) was used for detection as well as densitometry.

### Exposure of peroxynitrite donor to myofibrillar proteins

Male Wistar rats (9 weeks old, n = 4) were used for this experiment. Myofibrillar proteins were extracted from EDL muscles and incubated for 2 h at room temperature with the peroxynitrite donor 3-morpholinosydnonimine-N-ethylcarbamide (SIN-1) and the disulfide reductant dithiothreitol (DTT). The samples were then applied to immunoblots for actin and desmin as described above.

### 20S proteosome activity

Muscle pieces of approximately 80 mg were diluted in ice-cold homogenizing buffer (9 ul/mg wet wt) consisting of (mM): sucrose, 250; Tris/HCl, 50; MgCl_2_, 5; EGTA, 5; EDTA, 5; DTT, 1; ATP, 2; and 0.025% digitonin (pH 7.4). After centrifugation at 16,000 *g* for 15 min at 4°C, the resultant supernatant was collected and then protein concentration was determined as described above. Chymotrypsin-like activity of the 20S proteasome was measured using the assay of Kisselev and Goldberg [26]. N-succinyl-Leu–Leu-Val-Tyr-aminomethycoumarin (Suc-LLVY-AMC) served as a substrate. The homogenate was incubated for 10 min at 37°C in a buffer solution containing (mM): Tris/HCl, 50; KCl, 40; MgCl_2_, 5, DTT, 1; ATP, 0.5; and 0.5 mg/ml BSA (pH 7.4). The reaction was started by adding Suc-LLVY-AMC to give a final concentration of 25 μM and fluorescence of the liberated AMC was monitored in a fluorometer for 10 min (excitation 380 nm, emission 460 nm). Control assay was performed in the presence of 20 μM MG-132 (an inhibitor of proteasome and calpain) or 20 μM leupeptin (an inhibitor of calpain).

### Statistics

**Fig 1:**
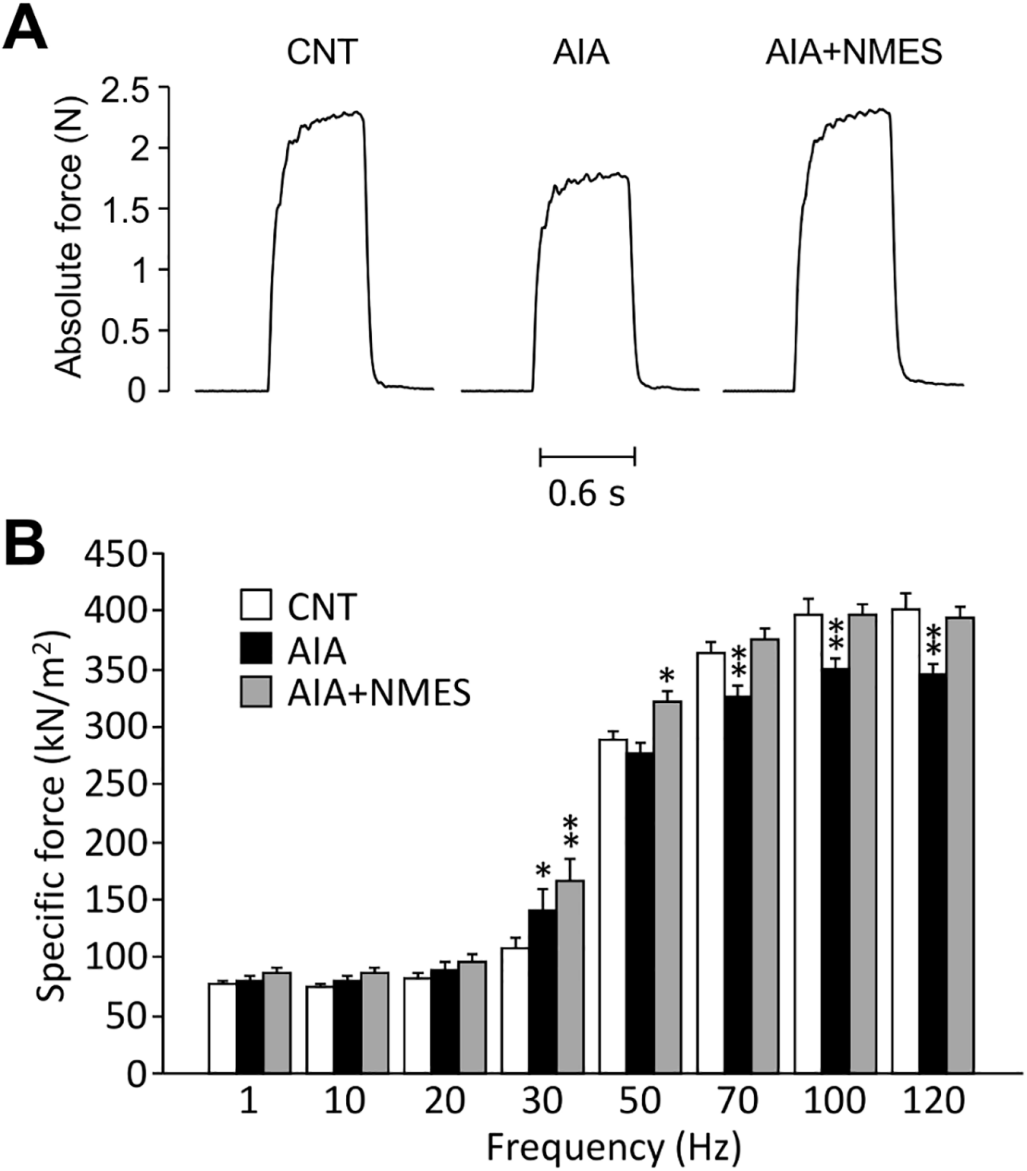
NMES training prevents contractile dysfunction in AIA EDL muscles. Representative original records of 120 Hz tetanic force in EDL muscles from control (CNT) and adjuvant-induced arthritis (AIA) rats with or without neuromuscular electrical stimulation (NMES) training (A). Specific force-frequency relationship (B). Bars show the mean and SEM results from 7–9 muscles per group. *P < 0.05, **P < 0.01 vs. CNT.

Data are presented as mean ± SEM. One-way ANOVA, or two-way repeated measures ANOVA (Fig 1 and S2 Fig), were used to test for differences vs. CNT. The Bonferroni *post hoc* test was used when ANOVA showed a difference vs. CNT. A *P* value less than 0.05 was regarded as statistically significant. Statistical testing was performed with SigmaPlot (version 13, Systat Software Inc, CA).

## Results

### NMES training does not change specific force of EDL muscles from normal rats

The average number of contractions was 37.3 ± 2.3 per session in *Experiment 1*. There was no difference in EDL muscle weights between CNT and CNT + NMES group (117 ± 3 versus 118 ± 4 mg (n = 6); *P* > 0.05). The specific force (i.e. force per cross-sectional area) did not differ between the CNT group and CNT + NMES group at any stimulation frequency (1–120 Hz; *see* S2 Fig). No further experiments were performed on these groups.

### NMES training prevents AIA-induced muscle weakness

In *Experiment 2*, AIA + NMES group received 37.0 ± 2.6 contractions per session. The body weight in AIA and AIA + NMES rats were significantly lower than those of the control group (Table 1). There was no difference in the EDL muscle weights between the groups. The maximum diameter of the knee joint was significantly higher (~20%) in AIA and AIA + NMES than in CNT rats, indicating that the extent of arthritis was not exacerbated by NMES training.

**Table 1:** Body weight, EDL muscle weight, and knee diameter of control and adjuvant-induced arthritis (AIA) rats.

	CNT (n = 8)	AIA (n = 9)	AIA+NMES (n = 7)
BWt (g)	303 ± 4	246 ± 4*	252 ± 5*
EWt (mg)	119 ± 2	111 ± 3	113 ± 2
knee (mm)	9.7 ± 0.1	11.6 ± 0.3*	11.5 ± 0.3*

Values are means ± SEM. CNT, control; AIA, adjuvant-induced arthritis; NMES, neuromuscular electrical stimulation; n, number of samples; BWt, body weight; EWt, EDL weight.

*P<0.05, compared with CNT.

Consistent with a previous study of our group [6], AIA induced contractile dysfunction in the EDL muscles (Fig 1A and 1B). Specific force was significantly lower in EDL muscles from AIA rats than that in CNT rats at stimulation frequencies from 70 to 120 Hz, and NMES training prevented this AIA-induced force reduction. Unexpectedly, specific force was higher in AIA and AIA+NMES muscles than in CNT muscles at 30 Hz, and it was also higher in AIA+NMES than in CNT muscles at 50 Hz.

### NMES training reduces the aggregation of myofibrillar proteins in AIA EDL muscles

**Fig 2:**
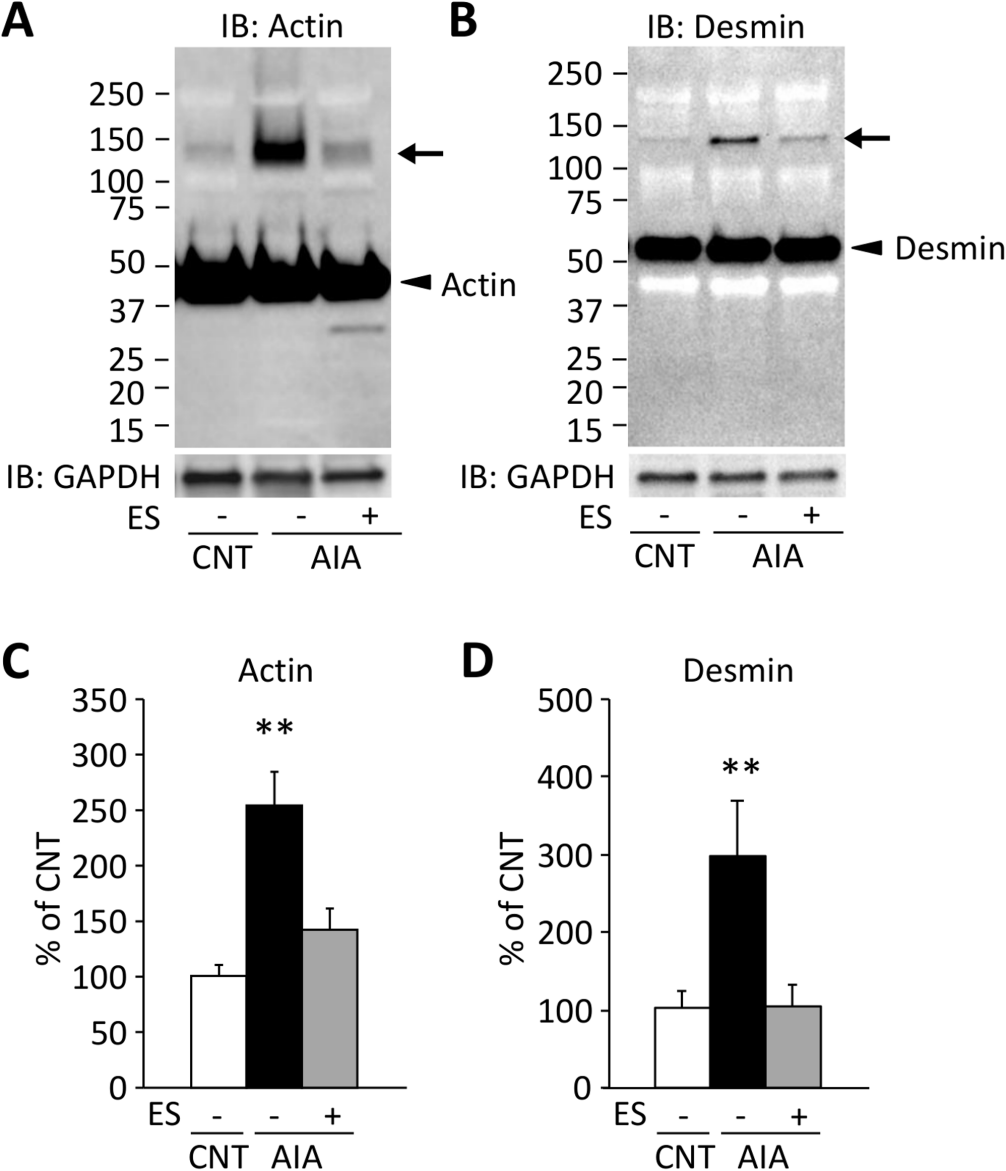
NMES training reduces the aggregation of myofibrillar proteins in AIA EDL muscles. Actin (A) and desmin (B) expression of EDL muscles in control (CNT) and AIA rats with or without neuromuscular electrical stimulation (NMES) training. The membranes were overdeveloped for the native proteins to visualize the ~130 kDa aggregates. The intensities for the protein band at ~130 kDa (indicated by an arrow) were normalized to the glyceraldehyde-3-phosphate dehydrogenase (GAPDH) content (C&D). Results are expressed as a percentage of CNT value. Bars show the mean and SEM results from 5–9 muscles per group. **P < 0.01 vs. CNT.

Actin aggregates were increased by 2.5 folds in AIA EDL muscles compared to the CNT muscles (Fig 2A and 2C). In addition to the actin aggregation, a desmin positive band was detected at a molecular weight corresponding to actin aggregates (Fig 2B) and the expression of this band was ~200% higher in AIA rats than in CNT rats (Fig 2D). These data suggest that AIA induces heterogeneous aggregation of myofibrillar proteins. Intriguingly, NMES prevented actin and desmin aggregation in AIA muscles (Fig 2C and 2D).

### Peroxynitrite donor induces actin and desmin aggregates in myofibrillar proteins from EDL muscles

**Fig 3:**
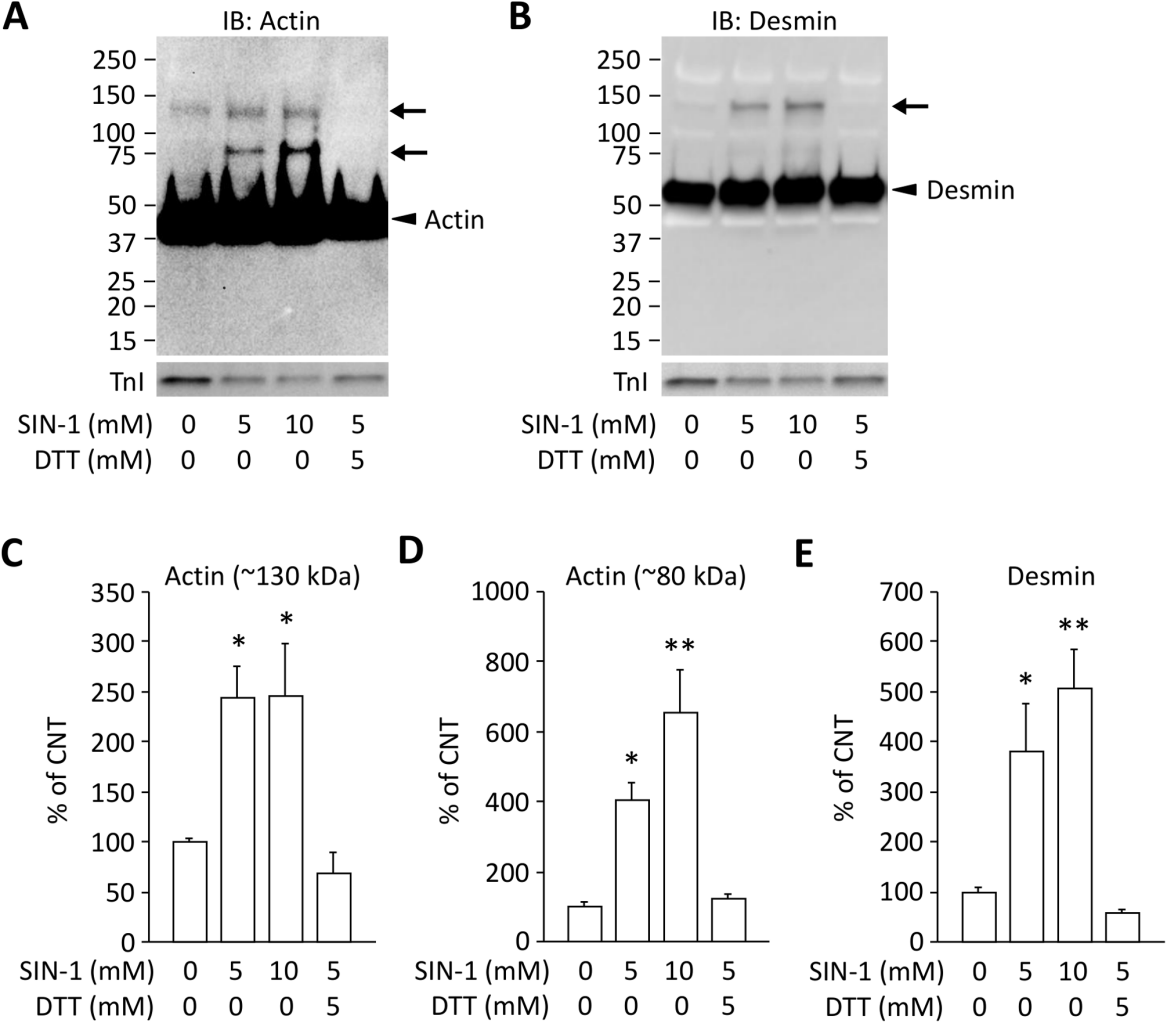
Peroxynitrite donor induces actin and desmin aggregates in myofibrillar proteins from EDL muscles. Actin (A) and desmin (B) expression of myofibrillar proteins in EDL muscles in the presence or absence of 3-morpholinosydnonimine-N-ethylcarbamide (SIN-1) and dithiothreitol (DTT). The membranes were overdeveloped for the native proteins to visualize aggregates. The intensities for the protein band at ~130 kDa (C) and ~80 kDa (D) for actin and ~130 kDa (E) for desmin (indicated by arrows) were normalized to the troponin I (TnI) content. Results are expressed as a percentage of CNT value. Bars show the mean and SEM results from 4 muscles per group. *P < 0.05, **P < 0.01 vs. CNT.

To investigate whether peroxynitrite is involved in the formation of protein aggregates, control experiments were performed where myofibrillar proteins from control EDL muscles were incubated for 2 h at room temperature with a peroxynitrite donor SIN-1. Immunoblots for actin and desmin showed the increased intensities for protein band at ~130 kDa in the presence of SIN-1, while immunoblot for actin also showed the increased intensities for protein band at ~80 kDa (Fig 3A–3E). Moreover, SIN-1-induced protein aggregates were prevented by DTT. Thus, these data show that peroxynitrite can induce disulfide bond-dependent actin and desmin aggregates.

### Expression levels of pro-oxidative enzymes, but not anti-oxidative enzymes, are increased in AIA EDL muscle

**Fig 4:**
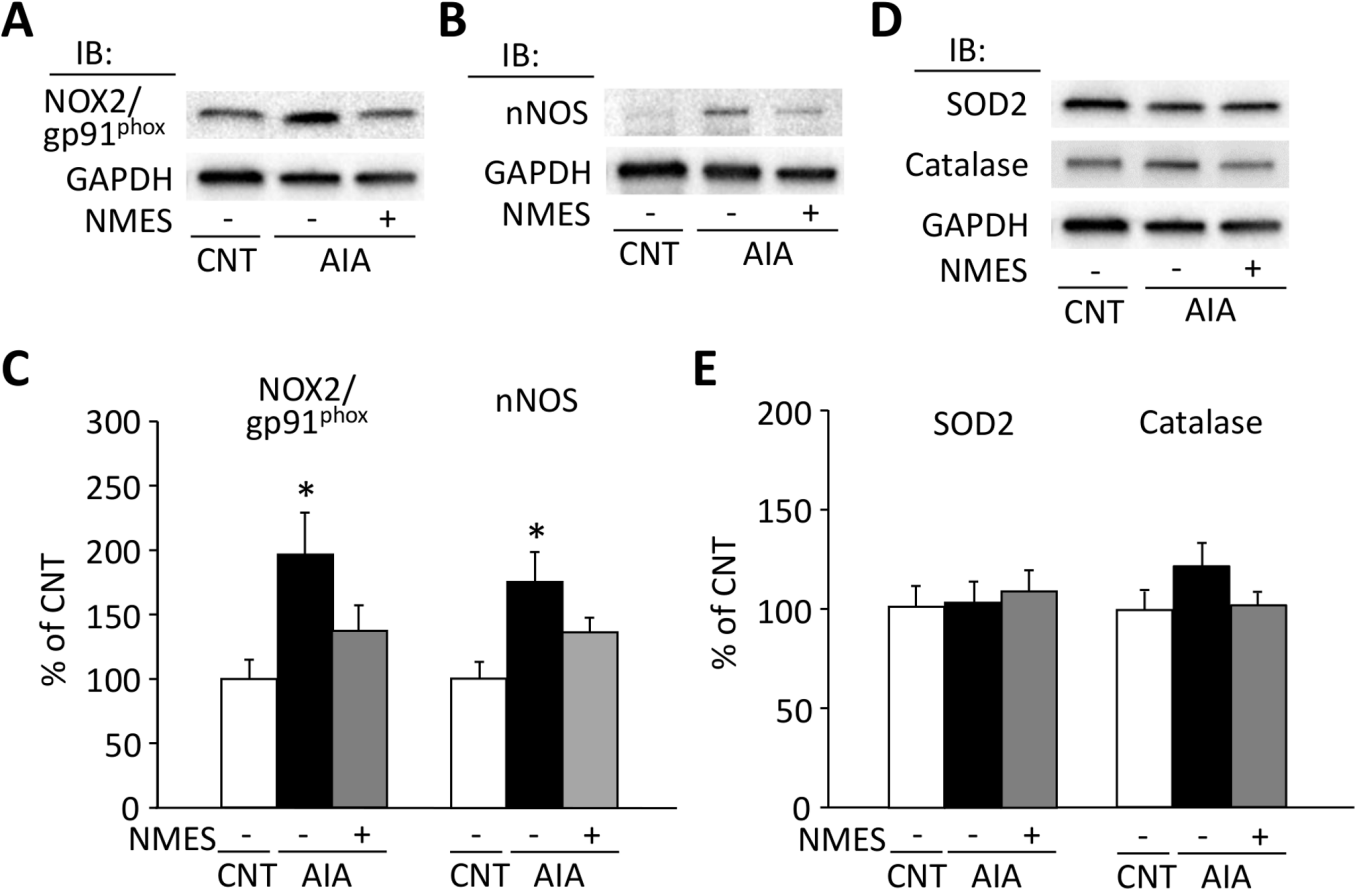
Expression levels of pro-oxidative enzymes are increased in AIA EDL muscle. Representative western blots illustrating the levels of NADPH oxidase (NOX2/gp91^phox^) (A), neuronal nitric oxide synthase (nNOS) (B), superoxide dismutase (SOD) 2, and catalase (D) of EDL muscles in control (CNT) and AIA rats with or without neuromuscular electrical stimulation (NMES) training. The levels of NOX2/gp91^phox^, nNOS (C), SOD2, and catalase (E) expression were normalized to the glyceraldehyde-3-phosphate dehydrogenase (GAPDH) content. Results are expressed as a percentage of CNT value. Bars show the mean and SEM results from 6–9 muscles per group. *P < 0.05 vs. CNT.

In agreement with our previous study [6], both NOX2 and nNOS expressions were higher in AIA than in control EDL muscles; NMES prevented these AIA-induced increases (Fig 4A–4C). The expression levels of SOD2 and catalase did not differ between the groups (Fig 4D and 4E).

### NMES training reduces the ubiquitination and restores the levels of autophagic marker in AIA EDL muscles

**Fig 5:**
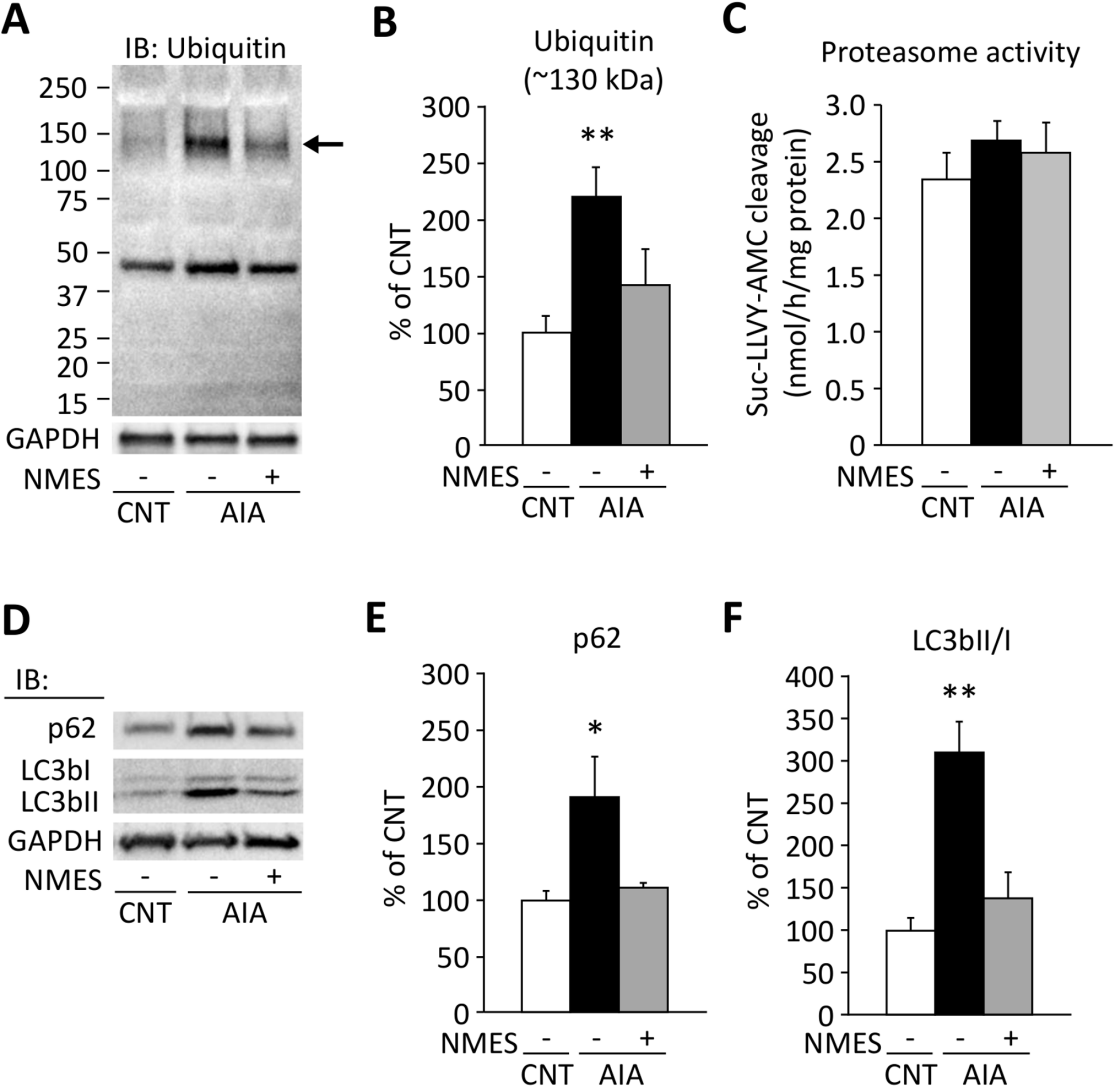
NMES training reduces the ubiquitination and restores the levels of autophagic marker in AIA EDL muscles. Representative western blots illustrating the levels of ubiquitinated proteins (A), p62, and LC3bI/LC3bII (D) of EDL muscles in control (CNT) and AIA rats with or without neuromuscular electrical stimulation (NMES) training. Intensities for the protein band at ~130 kDa (indicated by an arrow) in ubiquitinated proteins (B) and p62 (E) were normalized to the glyceraldehyde-3-phosphate dehydrogenase (GAPDH) content. The ratio of LC3bII/LC3bI (F). Results are expressed as a percentage of CNT value. Proteasome activity was similar in the three groups (C). Bars show the mean and SEM results from 6–9 muscles per group. *P < 0.05, **P < 0.01 vs. CNT.

We investigated whether impaired UPS is involved in the formation of myofibrillar aggregates in AIA EDL muscles. Intriguingly, increased ubiqutination was observed in the protein band at ~130 kDa where actin and desmin aggregates were detected in AIA muscles and NMES prevented the ubiquitination (Fig 5A and 5B).

Using synthetic fluogenic substrate, we next examined proteasomal peptidase activity. The chymotrypsin-like activity of the 20S proteasome was not altered in AIA EDL muscles (Fig 5C). These findings suggest that the primary defect is not the proteolytic core of 20S proteasomes but in the entry of ubiquitinated proteins into the 20S proteasomes.

An accumulation of the polyubiquitin-binding protein p62 has been regarded as the delay of autophagosome clearance. In contrast, the activation of autophagy drives the processing of LC3bI into its lipidated, autophagosome-bound form LC3bII, with a high LC3bII/LC3bI ratio being considered to indicate an increased autophagosome production. We observed an increase in the levels of the p62 and LC3bII/LC3bI ratio in AIA EDL muscles compared with CNT muscles (Fig 5D–5F), indicating the decreased clearance and the increased production of autophagosome. Interestingly, NMES restored these changes in AIA muscles.

## Discussion

Patients with chronic inflammatory diseases frequently suffer from muscle weakness that is, in part, independent of muscle atrophy [27]. Consistent with our previous study [6], we observed a reduction in specific force in EDL muscles from AIA rat. The force depression was present even after forces were normalized to the cross-sectional area, which indicates intrinsic contractile dysfunction in AIA EDL muscles. Decreased specific force production in skeletal muscle can, in principle, be attributed to reduced Ca^2+^ release from the SR, decreased myofibrillar Ca^2+^ sensitivity, and/or reduced ability of cross-bridge to generate force [28]. The force-frequency curve showed a force depression at high frequencies (i.e. 70–120 Hz), suggesting a problem with force generation capacity of cross-bridges in AIA EDL muscles, since both reduced SR Ca^2+^ release and decreased myofibrillar Ca^2+^ sensitivity have more impact on force at low frequencies because of the non-linear relationship between force and myoplasmic free [Ca^2+^][29].

Recently, we demonstrated that treatment with the antioxidant EUK-134 prevents the contractile dysfunction and oxidant-induced actin aggregation in EDL muscles from AIA rats [6]. These findings suggest that the formation of actin aggregates disrupts the myofibrillar function of muscle fibers in AIA EDL muscles. In the present study, we further investigated the underling mechanisms of arthritis-induced muscle dysfunction and found that desmin as well as actin are aggregated in AIA EDL muscles. Moreover, in vitro experiments showed that aggregation of these proteins can be induced by exposing myofibrillar proteins to the peroxynitrite donor SIN-1 (*see* Fig 3). The intermediate filament desmin has an important role in transmitting force and stabilization of sarcomere, and has been identified as a major target of redox modifications [30]. Mutations in desmin genes result in intracellular accumulation of desmin aggregates and impaired contractile function [30]. Notably, treatment with antioxidant was shown to prevent redox stress-induced desmin aggregation in muscle-like C2C12 cells with desmin mutations [31]. Thus, oxidation-induced actin and desmin aggregation can be the mechanisms behind the reduced specific force in AIA EDL muscles.

The present results demonstrate that NMES training prevents the reduction in specific force, the accumulation of actin and desmin aggregates, and the increase in NOX2 and nNOS in EDL muscles from AIA rats. Our findings of upregulation in NOX2 and nNOS suggest an increased production of peroxynitrite in AIA EDL muscles. In support, we previously showed increased levels of 3-nitrotyrosine, a protein modification produced by the reaction of peroxynitrite with tyrosine residues, in actin aggregates in AIA EDL muscles [6]. Thus, although the underlying mechanism is not clear, these data suggest that NMES prevents the AIA-induced increase in both NOX2 and nNOS, which counteract the overproduction of peroxynitrite. This then leads to inhibition of actin and desmin aggregates and prevents specific force depression in AIA EDL muscles. Interestingly, a previous study has shown that NOX2 inhibitor apocynin prevents contractile dysfunction and augmentation of 3-nitrotyrosine in diaphragm muscle from septic rats [32].

Endurance training has been shown to increase the expression level of SOD2 in rat soleus muscle [33]. Moreover, a single session of NMES training upregulated the mRNA of anti-oxidative enzymes, including SOD2 and catalase in rat soleus muscle [34]. In contrast to these studies, we could not see any changes in the amount of these anti-oxidative enzymes in AIA+NMES EDL muscles. The reason for this discrepancy is not clear. At any rate, it is unlikely that NMES training reduces the protein aggregates by augmentation of the anti-oxidative capacity in AIA EDL muscles.

Protein aggregation can be the result of impaired cellular proteolytic mechanism. The UPS plays a critical role in removing the denatured proteins from the cell. For degradation of UPS, the target protein molecule is first attached by a series of ubiquitin molecules. The ubiquitinated protein is then degraded by the 26S proteasome which consists of a 20S core and the 19S cap. Because ubiquitinated proteins were significantly increased and the peptidase activities of 20S proteasomes were not altered in AIA EDL muscles, the primary defect is a deficiency in the entry of ubiquitinated proteins into the 20S proteasome. Importantly, oligomeric or aggregated proteins are too large to enter the proteolytic core of the 20S proteasome, which may explain the increased ubiqutination at the molecular levels corresponding to actin and desmin aggregates in AIA muscles.

Autophagy is required for the removal of misfolded proteins and damaged organelles, and to prevent the accumulation of protein aggregates. In physiological conditions, a balance between autophagosome production and clearance maintains an adequate autophagic flux. In contrast, our results showed the activation of the autophagosome production, demonstrated by increased LC3bII/LC3bI ratio, and a delay in autophagosome clearance, demonstrated by the accumulation of p62, in AIA EDL muscles, suggesting an unbalanced autophagosome production/clearance ratio. These results correlates with increased levels of ubiquitinated proteins in AIA muscles. The importance of our findings stems from the fact that NMES promotes, not inhibits, the autophagic flux by a decrease in both LC3bII and p62 accumulation in AIA EDL muscles. In line with this, a recent study has reported that aerobic exercise normalizes autophagosome production/clearance ratio, which is associated with improved muscle function in tumor-bearing mice [17].

It has been reported that NMES-induced strength gains are positively correlated with the training intensity [35], and Adams et al. [23] showed hypertrophy of rat gastrocnemius muscle with supramaximal NMES training. In contrast, we used submaximal stimulation to maintain a peak torque corresponding to 60% of the maximal torque and observed no strength gain in normal rats. Although submaximal activation is routinely used for strength training protocols in human subject [36], we are not aware of any studies examining the effect of this training protocol on rat skeletal muscles. Thus, the stimulation intensity used in the present study may not be high enough to induce a strength gain in EDL muscles from normal rats. In contrast, it has been suggested that weakened muscle would respond more effectively to NMES [24], which fits with our results showing NMES-induced force gains in AIA muscles, but not in control muscles.

Unexpectedly, specific force was higher in AIA+NMES than in CNT muscles at 30 and 50 Hz, and it was also higher than CNT in AIA muscles at 30 Hz. The mechanism behind this unexpected increase of submaximal force is unclear. One tentative mechanism is increased SR Ca^2+^ release, which has previously been observed in muscle fibers of CIA mice [4, 5]. At 30–50 Hz stimulation, the force-frequency and force-Ca^2+^ relationships are steep and an increase in SR Ca^2+^ release can have a large force potentiating effect outweighing the depressed cross bridge force production. An alternative explanation for the higher forces at 30–50 Hz in AIA+NMES muscles might be training specificity, since NMES was performed at 50 Hz.

### Study limitations

Although the present results, together with our previous study showing positive effects with antioxidant treatment [6], provide clear-cut support for a causative link between redox-induced myofibrillar modifications and decreased force production in skeletal muscle of AIA rats, several conclusions regarding mechanisms are based on correlations. In relation to present study, pharmacological or genetic inhibition of NOX2 and/or nNOS at the time of AIA induction would more directly clarify the role of these enzymes in the development of muscle weakness and the beneficial effects of NMES. Moreover, the present experiments do not allow us to distinguish between the extra mechanical load or some other aspect of NMES as the trigger of beneficial effects in AIA muscles.

## Conclusions

The present study shows that AIA induces the contractile dysfunction in EDL muscles. This is likely caused by myofibrillar dysfunction due to the aggregation of actin and desmin. Notably, these deleterious alterations were prevented by NMES, presumably through preventing the increase of the pro-oxidative enzymes NOX2 and nNOS combined with restoring autophagy flux. Thus, our data implies that NMES training can be used to counteract muscle weakness in patients with RA.

## Supporting information

S1 Fig Procedure for neuromuscular electrical stimulation (NMES) training. Under anesthesia, the animal was placed in a supine position and the left limb was attached to a footplate connected to a force transducer. The foot was placed at 60°angle of plantar flexion. The peroneal nerve was stimulated using a pair of electrodes on the skin surface (A). Torque traces were displayed on a monitor (B), and the stimulation intensity was progressively increased throughout the stimulation period in order to maintain a peak torque corresponding to 60% of the maximum isometric torque (C).
(TIF)

S2 Fig NMES training does not alter specific force of EDL muscles from normal rats. Specific forces of EDL muscles in control (CNT) with or without NMES. Bars show the mean and SEM results from 6 muscles per group.
(TIF)
