# Supplementary figures and images for: Neuromuscular electrical stimulation prevents skeletal muscle dysfunction in adjuvant-induced arthritis rat
Source: PLoS One. 2017 Jun 21;12(6):e0179925. doi: 10.1371/journal.pone.0179925 (PMC5479592; doi:10.1371/journal.pone.0179925)

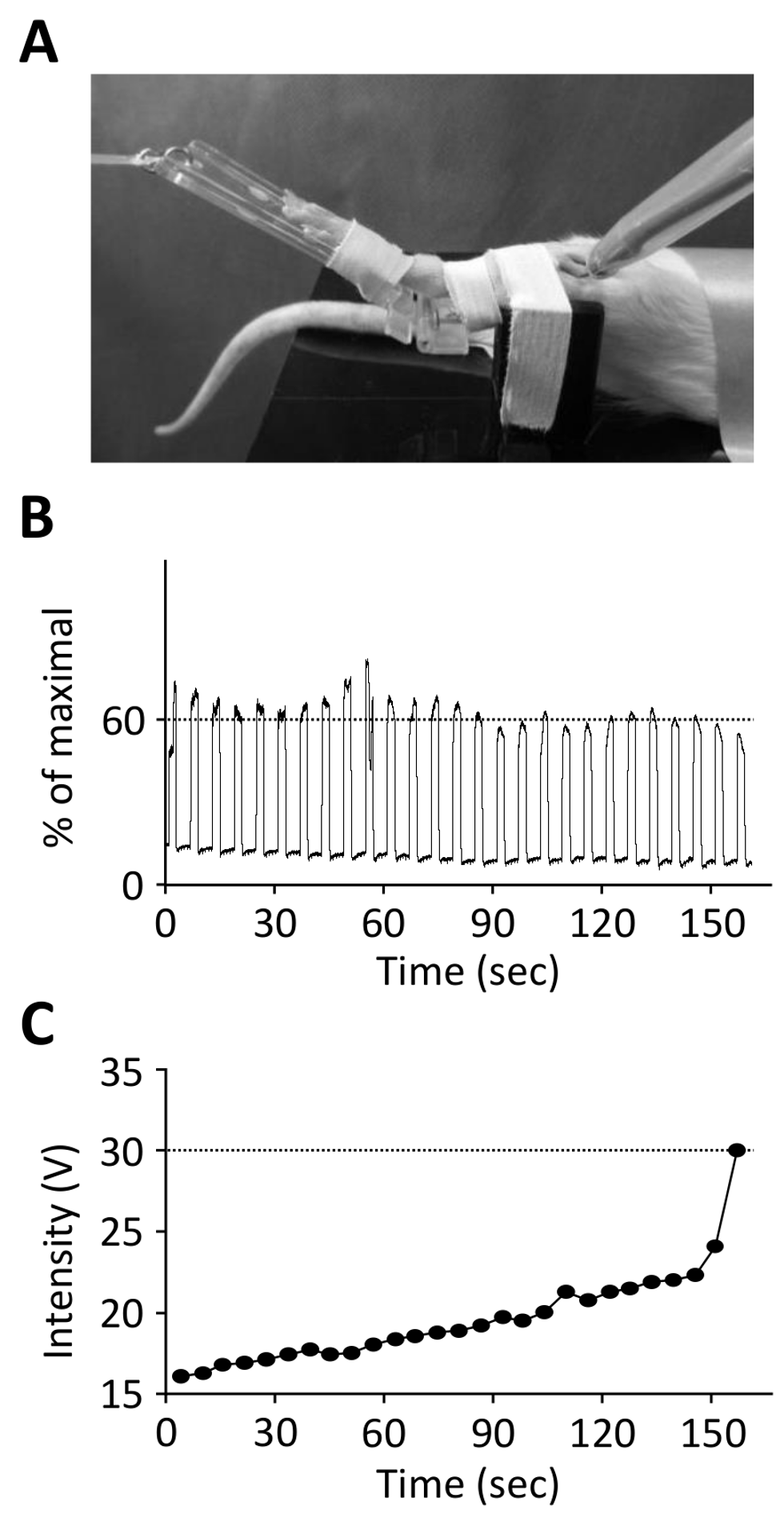

Supplement: S1 Fig — Under anesthesia, the animal was placed in a supine position and the left limb was attached to a footplate connected to a force transducer. The foot was placed at 60°angle of plantar flexion. The peroneal nerve was stimulated using a pair of electrodes on the skin surface (A). Torque traces were displayed on a monitor (B), and the stimulation intensity was progressively increased throughout the stimulation period in order to maintain a peak torque corresponding to 60% of the maximum isometric torque (C). (TIF) [file pone.0179925.s001.tif]

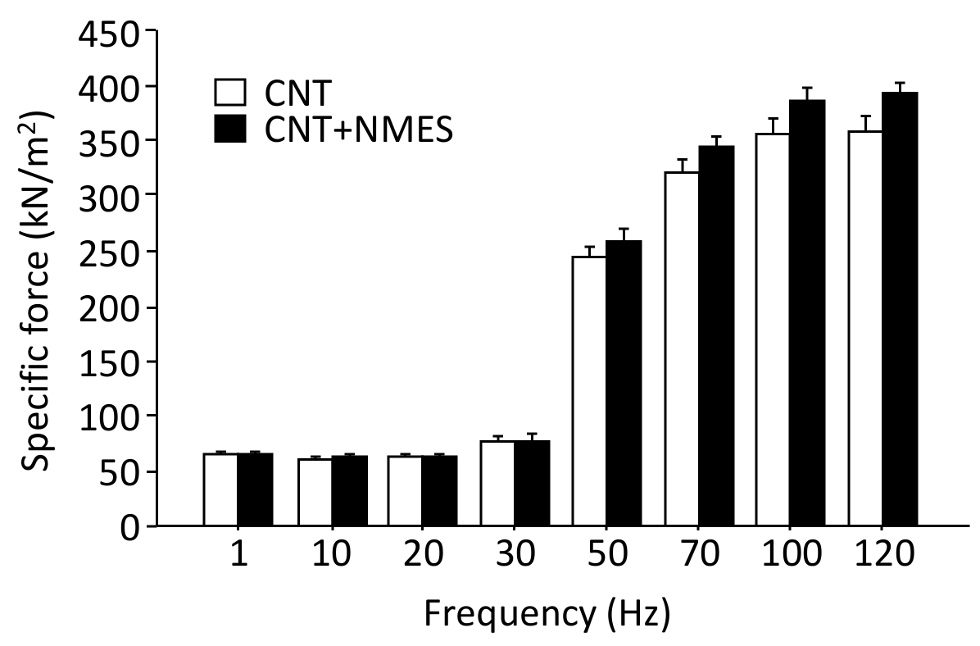

Supplement: S2 Fig — Specific forces of EDL muscles in control (CNT) with or without NMES. Bars show the mean and SEM results from 6 muscles per group. (TIF) [file pone.0179925.s002.tif]
